# Fluorescence in situ hybridization techniques for the rapid detection of genetic prognostic factors in neuroblastoma

**DOI:** 10.1054/bjoc.2000.1280

**Published:** 2000-06-02

**Authors:** C P F Taylor, N P Bown, A G McGuckin, J Lunec, A J Malcolm, A D J Pearson, D Sheer, on behalf of the United Kingdom Children's Cancer Study Group

**Affiliations:** Human Cytogenetics Laboratory, Imperial Cancer Research Fund, Lincoln's Inn Fields, London, WC2A 3PX, UK; Division of Human Genetics, Division of Pathology, Department of Histopathology, Department of Child Health, University of Newcastle-upon-Tyne, Royal Victoria Infirmary, Newcastle-upon-Tyne, NE1 4LP, UK

**Keywords:** prognosis, fluorescence in situ hybridization (FISH), *MYCN*, del(1p), ploidy

## Abstract

Neuroblastoma is the commonest extracranial solid tumour in children. There are a number of molecular genetic features known which are of prognostic importance and which are used to direct therapy. Identification and targeting of high-risk individuals with intensive therapeutic regimens may allow an improvement in survival rates. The most powerful biological parameters associated with prognosis in this malignancy are chromosomal changes, especially *MYCN* amplification, deletion of chromosome 1p and aneuploidy. Rapid characterization of these aberrations at the time of diagnosis is paramount if stratification according to risk group is to be achieved. This paper describes the rapid detection of del(1p), *MYCN* amplification and trisomy using interphase fluorescence in situ hybridization on imprints from fresh tumour biopsies. The results are related to those obtained by standard molecular methods and karyotyping. © 2000 Cancer Research Campaign


*British Journal of Cancer* (2000) **83**(1), 40–49
© 2000 Cancer Research Campaign
doi: 10.1054/ bjoc.2000.1280, available online at http://www.idealibrary.com on IDEAL®

### Fluorescence in situ hybridization techniques for the rapid detection of genetic prognostic factors in neuroblastoma

**CPF Taylor[1], NP Bown[2], AG McGuckin[3], J Lunec[3], AJ Malcolm[4], ADJ Pearson[5] and Denise Sheer[1], on behalf of the United Kingdom Children's Cancer Study Group**

[1]Human Cytogenetics Laboratory, Imperial Cancer Research Fund, Lincoln's Inn Fields, London, WC2A 3PX, UK; [2]Division of Human Genetics, [3]Division of Pathology, [4]Department of Histopathology, [5]Department of Child Health, University of Newcastle-upon-Tyne, Royal Victoria Infirmary, Newcastle-upon-Tyne, NE1 4LP, UK

**Summary** Neuroblastoma is the commonest extracranial solid tumour in children. There are a number of molecular genetic features known which are of prognostic importance and which are used to direct therapy. Identification and targeting of high-risk individuals with intensive therapeutic regimens may allow an improvement in survival rates. The most powerful biological parameters associated with prognosis in this malignancy are chromosomal changes, especially *MYCN* amplification, deletion of chromosome 1p and aneuploidy. Rapid characterization of these aberrations at the time of diagnosis is paramount if stratification according to risk group is to be achieved. This paper describes the rapid detection of del(1p), *MYCN* amplification and trisomy using interphase fluorescence in situ hybridization on imprints from fresh tumour biopsies. The results are related to those obtained by standard molecular methods and karyotyping. © 2000 Cancer Research Campaign

**Keywords:** prognosis; fluorescence in situ hybridization (FISH); *MYCN*; del(1p); ploidy

Neuroblastoma, a tumour arising from embryonal neuronal tissue, accounts for 10% of all cancers in those under 15 years old with an annual incidence of 6–8 per million. In the past it was always fatal for children with metastatic disease over 12 months old. Now a better understanding of the disease, with more sophisticated investigations and new therapies, has improved the 2-year survival rate of children with advanced disease to 25%.

Accurate stratification of patients at diagnosis into different prognostic groups is possible using various univariate methods which relate clinical and biological factors to outcome (Evans et al, 1987; Oppedal et al, 1988). Age at diagnosis is often considered to be the most important independent clinical prognostic factor. In 1984, Shimada et al described age-dependent histological criteria which have unequivocal bearing on prognosis.

Poor prognosis has been found to be associated with a number of biochemical markers. However, chromosomal changes in neuroblastoma are probably the most powerful biological factors associated with prognosis in this disease. The most significant are *MYCN* amplification, deletions at 1p36 and aneuploidy.

The *MYCN* oncogene was first identified in 1983 and later localized to 2p23–24 (Schwab et al, 1983, 1984). Amplified *MYCN* is frequently in the form of homogeneously staining regions found at varying chromosomal loci, but not at 2p23–24. Amplification is present in 38% of stage 3 and 4 tumours (Brodeur et al, 1984) and only 5–10% of patients with stage 1,2 or 4S disease. (Brodeur and Fong, 1989). In many patient cohorts *MYCN* amplification has been found to be associated with rapid tumour progression and a poor outcome irrespective of the stage of the tumour (Seeger et al, 1985; Tsuda et al, 1987; Taylor and Locker, 1990; Bourhis et al, 1991*b*; Look et al, 1991). Thus, in those with early-stage tumours, which generally have a good prognosis, *MYCN* amplification is an indicator of aggressive disease. Stage 4S, which is characterized by frequent spontaneous regression without therapy, rarely shows amplification (Ambros et al, 1995). Therapy is currently being directed according to *MYCN* gene amplification. In infants with neuroblastoma and patients with localized disease this guides their treatment.

Deletion of chromosome 1p is an independent indicator of poor prognosis (Christiansen and Lampert, 1988; Hayashi et al, 1989) and may be the most discriminant prognostic factor (Caron et al, 1996; Rubie et al, 1997). Early studies showing deletion in 70% of samples concentrated on advanced tumours and cell lines (Brodeur and Fong, 1989; Weith et al, 1989), which were likely to be a non-representative group of aggressive, progressive tumours. Del(1p) is less often found in stage 1 and 2 tumours (Hayashi et al, 1989; Ambros et al, 1995). Assessments of loss of heterozygosity (LOH) on chromosome 1p using polymorphic DNA markers, restriction fragment length polymorphism (RFLP), polymerase chain reaction (PCR) and single-strand conformation polymorphism (SSCP), have produced estimates ranging from 10 to 37% (Fong et al, 1989; Peter et al, 1992; Caron et al, 1993, White et al, 1993; Schleiermacher et al, 1994; Takeda et al, 1994; Rubie et al, 1997).

DNA ploidy is a major prognostic factor in neuroblastoma. In 1984, Look et al found that aneuploid tumours responded well to chemotherapy. Flow cytometric studies correlated ploidy with histological prognostic factors and clinical outcome, and confirmed this (Gansler et al, 1986; Taylor et al, 1988). Absence of

*Received 24 February 1999*
*Revised 14 February 2000*
*Accepted 24 February 2000*

*Correspondence to:* CPF Taylor, Department of Haematology, Royal Free Hospital, Pond Street, London NW3 2QG, UK

aneuploidy (a near diploid or near tetraploid DNA content), is associated with more aggressive disease. Aneuploid, or near triploid, karyotypes are extremely unlikely to have *MYCN* amplification, whereas a subset of those without aneuploidy do have *MYCN* amplification (Taylor and Locker, 1990; Bourhis et al, 1991*a*, 1991*b*). Look et al concluded that in children under 2 years old tumour cell ploidy and *MYCN* copy number provide complementary prognostic information on which future treatment strategies may be based (Look et al, 1984, 1991).

Fluorescence in situ hybridization (FISH) and comparative genomic hybridization (CGH) studies have recently established that gain of chromosome 17 material is the most frequent abnormality in neuroblastoma cells (Meddeb et al, 1996; Lastowska et al, 1997; Bown et al, 1999). Unbalanced gain of the long arm of chromosome 17 is associated with other characteristics of poor prognostic significance (age under 1 year, 1p loss, *MYCN* amplification and adverse ploidy levels). The molecular pathology underlying 17q gain remains to be elucidated, but recent studies suggest that it is a feature of considerable prognostic significance (Bown et al, 1998).

Standard techniques are effective for detection of prognostic chromosome changes in neuroblastoma but may be impractical owing to the time taken to perform the test, the quantity of tissue required and other confounding factors. Southern blotting requires more tumour tissue than FISH and results may be adversely affected by admixture of normal tissue in the sample (Shapiro et al, 1993). Karyotyping produces a high rate of del(1p) because the abnormality itself confers a growth advantage in culture (Christiansen et al, 1992).

In this study we employed interphase cytogenetic techniques using FISH on tumour imprints from fresh biopsy material. We previously evaluated this technique on control fibroblasts and neuroblastoma cell lines as well as six primary tumour samples (Taylor et al, 1994). We have now studied 58 patients samples using probes for *MYCN*, 1p36 and centromere probes for ploidy.

We have applied these rapid simple techniques which circumvent the difficulties of culturing and karyotyping, and of molecular techniques, allowing the results to be available to clinicians within a few days of receipt of a fresh tissue biopsy.

### MATERIALS AND METHODS

### Materials

The plasmid probe for *MYCN*, pNb-9, consists of a genomic *Hind*III fragment of 15 kb in pBR322. The chromosome 1 centromere probe used was pUC1.77, a satellite III repetitive DNA probe located in the heterochromatic region of chromosome 1 (1q12) (Cooke and Hindley, 1979). CT4-1 is a 47-kb cosmid clone which was isolated from a library using a plasmid subcloned DNA probe p1–24, which maps close to the consensus deletion at 1p36.1–2 (Weith et al, 1989). A centromere probe p4.4 for chromosome 8 was used as a control and to obtain additional information about ploidy.

Human foreskin fibroblasts (HFF) were used throughout as a normal control and neuroblastoma cell lines PCF, IMR32, Kelly, GOTO, SK-N-BE, were used as controls for *MYCN* amplification.

Neuroblastoma samples were obtained via the UK Children's Cancer Study Group (UKCCSG) and the European Neuroblastoma Study Group (ENSG). Fresh tumour biopsies were initially sent in culture medium (RPMI-1640) by courier as soon after removal from the patient as possible. Later tumour imprints on glass slides, made by the histopathology staff at the hospital of origin were sent dried but unfixed by ordinary mail. Other samples received included bone marrow smears, cytospins and a fine needle aspirate.

### Methods

#### *DNA probes*

Plasmid and cosmid DNA preparation was carried out according to standard mini-prep and maxi-prep techniques. The DNA used to screen cosmid libraries was prepared from plasmid p1–24. Ten-millilitre cultures were grown overnight with ampicillin and DNA was isolated using the mini-prep procedure.

#### *Cell culture and harvesting*

HFFs were cultured in Dulbecco's minimal essential medium (DMEM) at 37°C supplemented with 10% fetal calf serum (FCS) and 1% glutamine with 10% carbon dioxide ($CO_2$). The neuroblastoma cell lines were cultured in DMEM with 10% FCS and 1% non-essential amino acid supplement at 37°C with 10% $CO_2$. Direct nuclear preparations were prepared from fresh biopsy samples of both types of tumour by mincing with scissors to produce a single cell suspension as previously described (Taylor et al, 1994).

#### *Slide preparation*

Imprints were made directly onto clean, non-coated slides using a dry, blood-free, newly cut surface of fresh, unfixed biopsy material. This was done as soon as possible after removal from the patient, either at the hospital of origin, or after transport in tissue culture medium (RPMI-1640) to the laboratory. Six imprints were made of each specimen. Dry slides were then fixed and cleaned of debris using glacial acetic acid (Taylor et al, 1994).

Glass microscope slides were wiped clean with fixative before use with cultured cells and direct nuclear preparations. The cell suspension was dropped onto the slide from a pipette and left to dry. The density of the cells on the slides was checked using phase microscopy and adjustments made by the addition or removal of fixative.

Cytospin preparations were made at the hospital of origin directly onto clean glass slides. Fluid obtained by fine-needle aspiration was spread directly on clean slides at the referring hospital, and left to dry in air. Ascitic fluid was spun at 300 ***g***, suspended in potassium chloride (KCl) and then fixed as above before slide making.

Slides were artificially aged by baking at 65°C for 2–4 h prior to use to facilitate short turnaround times. Otherwise 1 week storage at 4°C gave equally good results. All slides could be stored in a 4°C refrigerator for up to 6 weeks, or for longer at –40°C with a desiccant if required.

#### *FISH methods*

The probes for all experiments were labelled by nick-translation with biotin-11-dATP (BRL Bio-nick kit) or else with digoxygenin-11-dUTP (Boehringer, Mannheim, Germany) according to the suppliers' instructions. The probes were purified through a Sephadex G50 column and precipitated with salmon sperm DNA and *Escherichia coli* tRNA.

Hybridization and detection were performed according to our modification of the technique described by Pinkel et al in 1986 (Taylor et al, 1994).

© 2000 Cancer Research Campaign

Each of the four probes used was hybridized onto a separate slide. If two types of preparation were available for an individual patient then all four probes were hybridised to both sets of slides.

The neuroblastoma cells in these preparations were frequently single, but often there was nuclear clumping. Clumped nuclei were included in the study, but overlapping nuclei were excluded. On some slides red blood corpuscles were still present, which take up fluoroscein isothiocyanate (FITC) non-specifically, so that when they were overlapping a tumour cell that cell had to be excluded. The signals counted in each nucleus were of equal intensity to each other, though there was slight variation from cell to cell. Minor hybridization spots and background fluorescence were discounted. Signals had to be completely separate from one another to be included; paired spots close together were counted as one signal.

Inevitably there was an admixture of tumour cells and normal stromal or haemopoietic cells on each slide, which varied from one area to another. It was meaningless, therefore, to include a pre-set number of random nuclei and calculate average signal numbers. Only the cells which were considered most likely to be tumour cells, after parallel morphological examination of a May–Grunwald–Giemsa (MGG) stained slide of the same preparation, were included. In fact the proportion of non-tumour cells in the tumour imprints was very low (< 15%) whereas in some bone marrow smears it was considerably higher. However, there was no difficulty in identifying which cells to include.

In all samples there were nuclei which did not react with the DNA probes and also there were infrequent cells with three or more signals. Control experiments were carried out with both centromere probes, CT4-1 and pNb-9 using HFF nuclei and the PCF cell line, examining 600 nuclei for signal number. In the tumour imprints and bone marrow smears at least 50, and preferably 100, nuclei were examined which were believed to be of tumour origin. The hybridization was repeated using more probe DNA in those where < 70% of nuclei showed a consistent result. In practice a small proportion of the cases reported below required a repeat hybridisation for CT4-1 in order to achieve this result. (See below for results of control experiments.)

The inclusion of the chromosome 8 centromere probe meant that those cases with three or four copies of chromosome 1 could be more securely classified as triploid or tetraploid rather than merely trisomic or tetrasomic for chromosome 1. Chromosome 8 was selected from a panel of possible centromere probes because it is not specifically duplicated or deleted in neuroblastoma, and because the probe available is reliable and specific.

### RESULTS

Control experiments using FISH were carried out on normal HFF cells and neuroblastoma cell lines. The presence of del(1p), *MYCN* amplification and tumour cell ploidy level were determined by the application of FISH to various preparations of neuroblastoma samples, mainly tumour imprints. Where feasible genetic data were also obtained by Southern blotting (*MYCN*) and conventional karyotyping.

### Control experiments on normal HFF nuclei and neuroblastoma cell lines

The sensitivity of the chosen probes was assessed by applying them to preparations of normal HFF nuclei and examining 600 nuclei. Imprints of comparable normal tissue, or bone marrow smears containing chromosomally normal but morphologically identifiable non-haemopoietic cells were not readily available for comparison. Ten neuroblastoma cell lines were evaluated by karyotyping and chromosome painting in order to select those most apposite as controls (Taylor et al, unpublished data).

#### *Centromeres*

The chromosome 1 centromere probe, pUC1.77, produced two clear separate signals in 78% of normal nuclei. In addition, 2% showed no signal, 14% showed one signal, 3% three signals and 3% four signals. The second centromere probe, p4.4 (chromosome 8 centromere), which was selected both as a control and to help make an estimate of tumour cell ploidy, produced two signals in 83% of normal nuclei. Proportions for no signal, one, three and four signals were 0%, 10%, 2% and 5% respectively.

#### *Probe for del(1p)*

Using the distal 1p probe, CT4-1, 80% of normal HFF nuclei displayed two hybridization signals, < 1% no signal, 5% one signal, 4% three signals, 9% four signals and 2% > 4 signals. CT4-1 was also hybridized onto interphase preparations of the neuroblastoma cell line PCF. Two signals were clearly visible in approximately 80% of nuclei. This was the expected result because this line has four copies of chromosome 1, two of which have distal 1p deletions, leaving two intact copies to which the terminal 1p probe will hybridize.

#### MYCN *amplification*

Using the *MYCN* probe pNb-9, two discrete signals were seen in 84% of HFF nuclei. In addition nuclei from four neuroblastoma cell lines known to have amplification of *MYCN* were prepared. IMR32 has approximately 20 copies of *MYCN* per cell, GOTO has 60 copies, SK-N-BE has 150 copies and Kelly 120 copies per cell. This produces a very characteristic appearance under the fluorescence microscope which differs from background fluorescence. Signal numbers in interphase nuclei were then counted in broad categories: 0–20; 20–50; 50–100 and > 100. A median value was therefore taken in each case. Wide cell to cell variation in copy number was observed in the primary tumours with *MYCN* amplification probably due to random segregation of DMs during mitosis.

In order to obtain an estimate of the number of copies in the patient samples, signals were counted as accurately as possible at microscopy in 100 nuclei and then divided into categories as above depending on the number of *MYCN* signals seen. A median *MYCN* content for each tumour sample was then taken. Frequently two or three discrete signals from the *MYCN* probe were seen in the nuclei of the cases in which the gene was not amplified.

### Results on patient samples using FISH

Results were analysed on a total of 68 samples, which were performed on a total of 58 patients. Six of the ten patients for whom more than one sample was tested had both direct nuclear preparations and tumour imprints available from the same biopsy, and a further one had tumour imprints and bone marrow slides from biopsies taken at the same time. Two had pre- and post-chemotherapy samples (one bone marrow and one imprints) and one had a bone marrow at the time of presentation and again at relapse.

 © 2000 Cancer Research Campaign

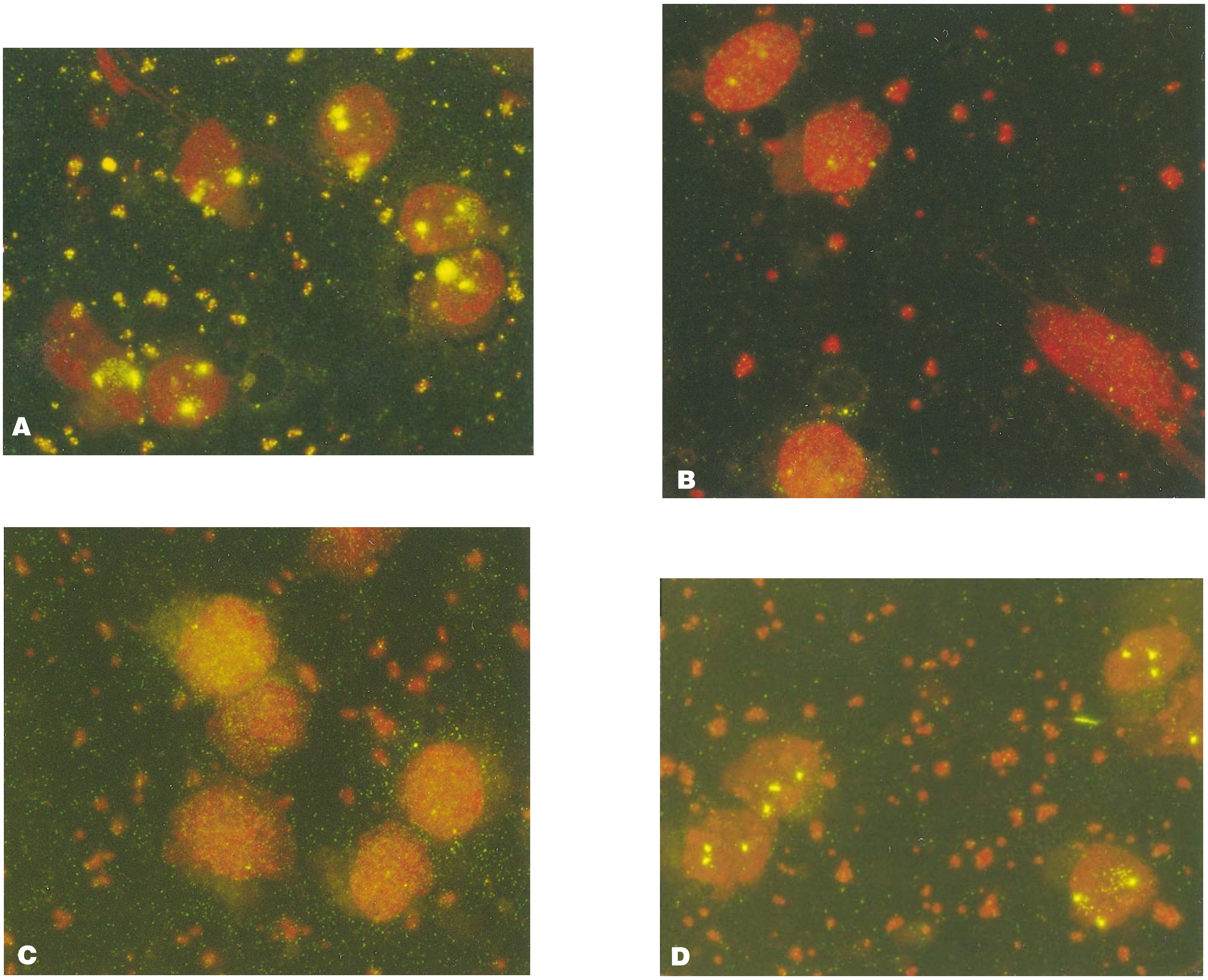

**Figure 1** (**A**) Chromosome 1 centromere; (**B**) CT4-1 (distal 1p probe); (**C**) *MYCN* probe; (**D**) chromosome 8 centromere. Typical appearance of the four selected probes on a tumour imprint slide of average quality (case 5). There is debris on the slide which sometimes picks up fluorescence, and the chromosome 1 centromere probe occasionally gives a slightly diffuse signal, but interpretation of the signals is not affected. There are two copies of chromosome 1 centromere and CT4-1 and three copies of chromosome 8 centromere. *MYCN* is not amplified

Of the 68 experiments 52 gave a full set of FISH results (75%). A further five samples were only interpretable for *MYCN*, thus producing a total of 56 experiments (82%) which yielded clinically important prognostic information.

The majority of experiments (63%) were performed on tumour imprints which gave an 81% success rate, while experiments on marrow smears (10% of the total) and direct preparations (also 10%) were 100% successful. Cultured cells were used for five patients (7%) and all yielded a full set of results. However, all cultured cells were normal diploid with no *MYCN* amplification. It is likely that only normal fibroblasts were present in the culture, a fact borne out by the frequency of the same problem with conventional cytogenetic techniques. A fine-needle aspiration (FNA), a cytospin and an imprint from previously frozen tissue were found to be unsuitable for FISH. The FNA had too few cells for a meaningful assessment to be made; the cytospin produced overcrowded cells with indistinct borders so that it was unclear which signals arose from which cell; and the previously frozen tissue was full of necrotic debris and the signals were very few and faint. Further attempts to obtain results with these three types of preparation were not made as the problems encountered were very likely to be recurrent.

If the three unsuitable preparations, and the five cultured samples are subtracted from the total number of experiments performed, this leaves a total of 60 experiments in which one could reasonably expect to obtain interpretable, meaningful results. Of these 51 (85%) gave results. These 60 experiments were carried out on 50 different patients (there were ten patients who had two samples, as described above). A full set of FISH results was obtained on 38 of the 50 patients (76%) and full or partial results on 42 (84%).

The findings in these 38 patients were as follows: diploid without *MYCN* amplification or del(1p) – ten patients (26%); triploidy without *MYCN* amplification or del(1p) – 13 patients (34%); Del(1p) without *MYCN* amplification, diploid – four patients (11%); *MYCN* amplification and del(1p), diploid – six patients (16%); Other findings, e.g. complex karyotypes with out del(1p) or *MYCN* amplification – five patients (13%). A series of photographs showing the results of a selection of the FISH experiments on the above patients are presented in Figures 1–4.

*© 2000 Cancer Research Campaign*

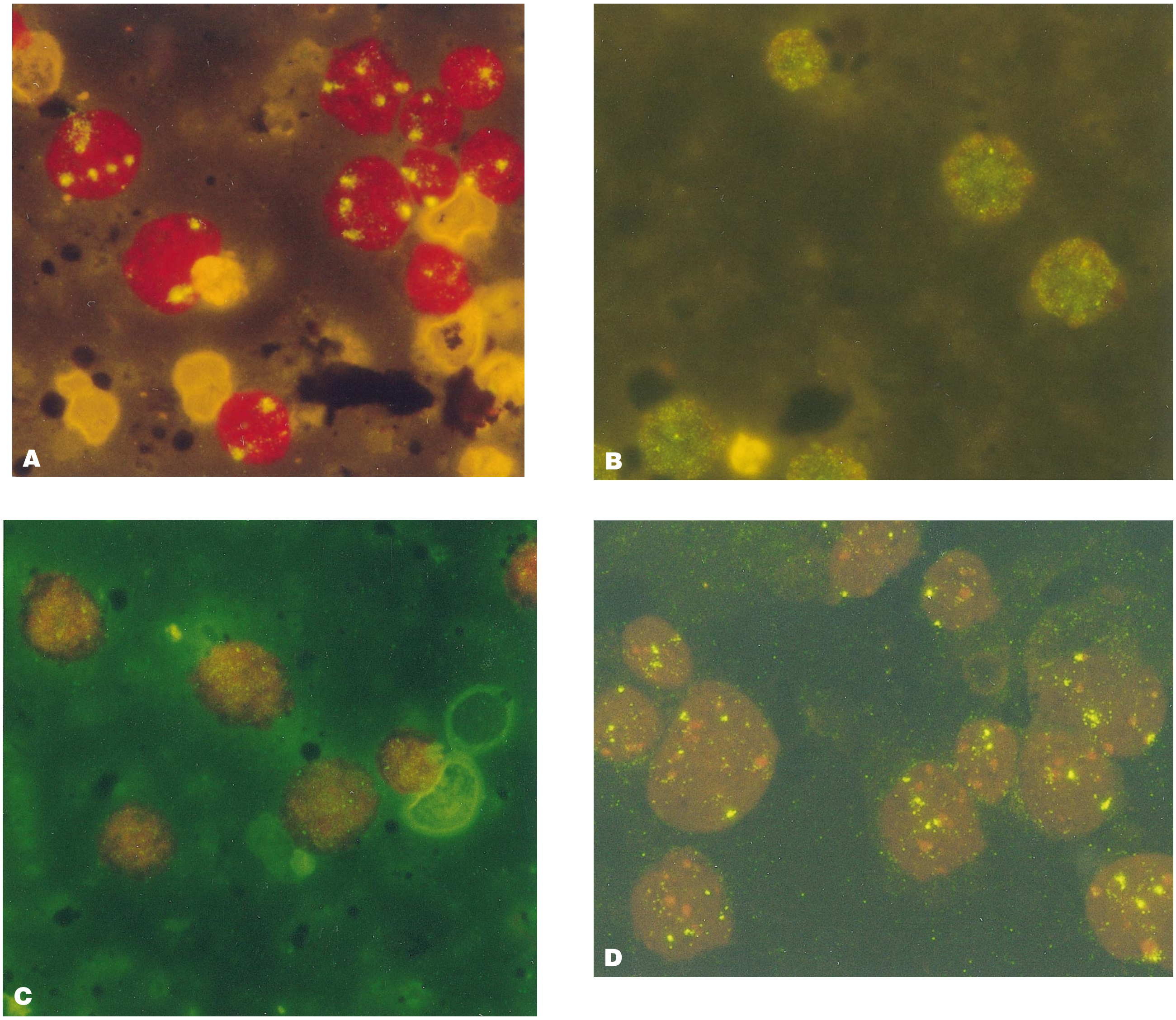

**Figure 2** (**A**) Chromosome 1 centromere; (**B**) CT4-1 (distal 1p probe); (**C**) *MYCN* probe; (**D**) chromosome 8 centromere. Bone marrow smear showing two populations of cells (case 29). Twenty-five per cent are probably tetraploid cells and the rest are diploid (centromere probes 1 and 8). The large cells show a maximum of three copies of CT4-1 while the diploid cells have two copies, suggesting that there is a 1p deletion in the tetraploid population but not the diploid population. *MYCN* is not amplified, with single copies of this small probe being visible in some cells

### Correlations of FISH results with results from standard techniques

### *Southern blotting*

Biopsy samples were sent at presentation for Southern blotting in 32 (55%) of the the total of 58 patients in the study. The number of individual patients (as opposed to the total sample number) is used here because duplicate bone marrow samples were not used for Southern blot techniques, and follow-up samples were not requested. Blotting results were obtained on 27 of 32 samples (84%), with the five failures being due to insufficient or poor quality DNA from small samples.

There are 22 patients on whom there are both FISH results and Southern blot results for *MYCN*, and of these there is correlation of findings in 21 (95%). The one discrepancy is on a sample early in the study in which FISH results were misinterpreted as positive for *MYCN*, but the Southern blot was negative.

### *Conventional karyotyping*

There were 62 different samples on which karyotyping was a possibility, as the total of 68 samples included six cases where direct cell suspension and imprint were made from the same biopsy. Karyotyping was attempted on 30 (48%) of the 62 samples, and failed in five (17%), while a further 12 (40%) were only successful in long-term culture where both phase microscopy and the consistently normal results indicated that only fibro blasts remained in the culture. One sample was sent but not processed, and one was inadequate. Full or partial karyotyping results were obtained in 11 (37%) of the 30 attempted. In three cases only an estimate of ploidy and account of the approximate number of marker chromosomes were possible because of poor qualitychromosomes. In a further three cases two copies of chromosome 1 could be identified, although a complex hyperdiploid karyotype meant that the presence of further abnormal copies of chromosome 1 could not be excluded.

 © 2000 Cancer Research Campaign

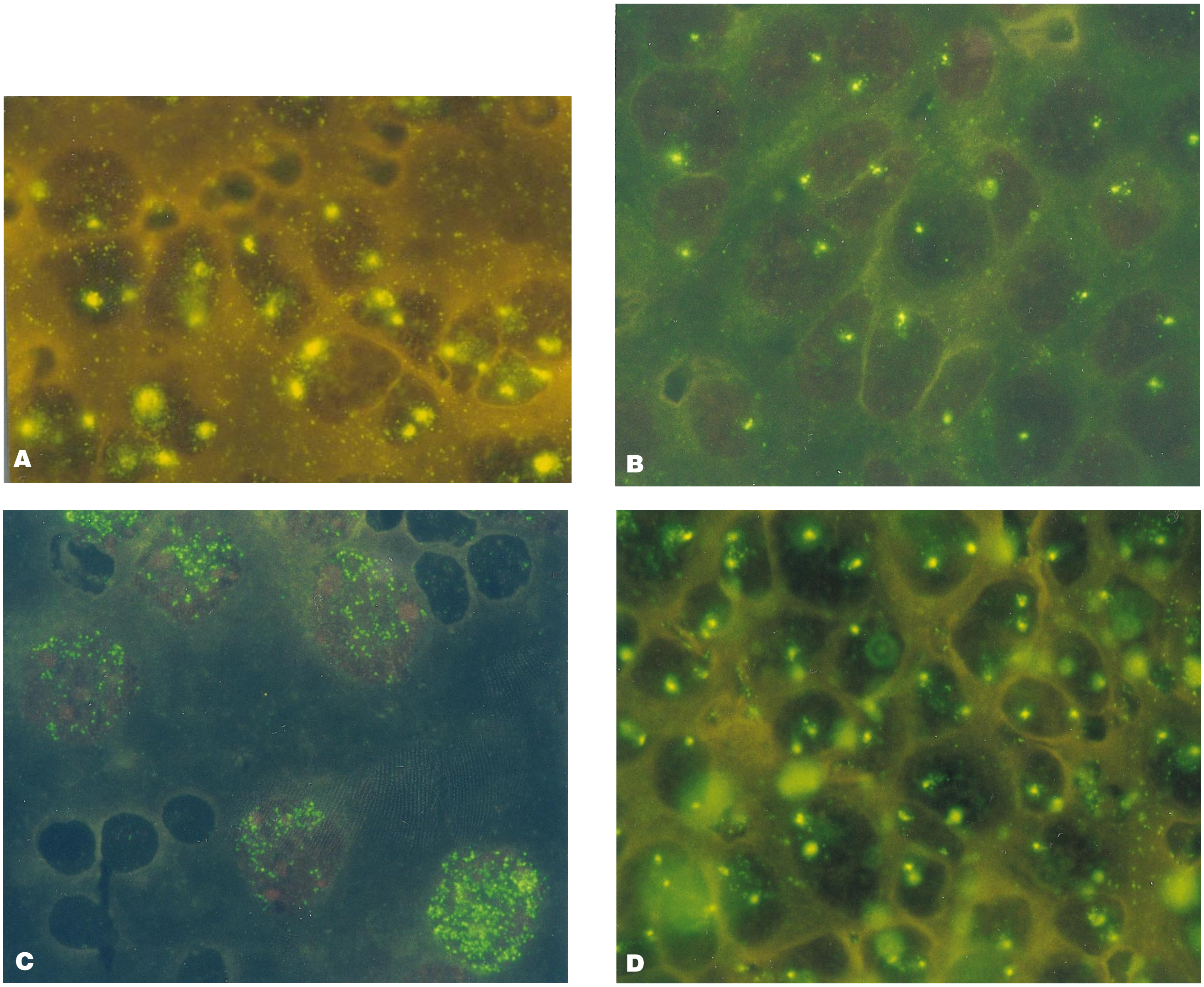

**Figure 3** (**A**) Chromosome 1 centromere; (**B**) CT4-1 (distal 1p probe); (**C**) *MYCN* probe; (**D**) chromosome 8 centromere. Imprint of a patient's tumour with del(1p) and *MYCN* amplification (case 52). This imprint is very crowded with tumour cells and debris, a common finding, but the results are still perfectly clear. There are two copies of both centromere probes so the cells are likely to be diploid. The distal 1p probe CT4-1 is present in only one copy per cell, providing definite evidence of a 1p36 deletion, and *MYCN* is clearly amplified with over 100 signals per cell

This leaves five samples (17% of those attempted) for which a full karyotype was obtained. Two of these were from the same patient but were different samples (one tumour biopsy and one bone marrow) taken at the same time and producing duplicate results.

Ten of the eleven cases with karyotyping results also had results using FISH. (The exception was case 12 on whom only frozen tissue had been available for FISH.) The 10 cases with both karyotyping results and FISH results showed a full correlation of results in all cases. In four patients a full karyotype was available (one bone marrow aspirate and three solid tumour biopsies), and of these one had a del(1p) and the remaining three did not, which correlated fully with FISH results. In three of the six samples in which there were incomplete karyo typing results, complex rearrangements were detected by both methods, but del(1p) was detected by FISH in some cell populations, while karyotyping was limited to visualisation of marker chromosomes.

### DISCUSSION

The aim of this work was to develop simple, rapid and readily reproducible tests, for the presence of the clinically significant genetic aberrations found in neuroblastoma. This technique produces reliable clinically relevant results on a small tissue sample and may have a role in a national laboratory in which data on all neuroblastomas can be collated. It is the intention of the ENSG to cooperate with such national laboratories in various European countries.

Overall this study found 26% of neuroblastomas were diploid without amplification of *MYCN* or del(1p); 34% were apparently triploid without *MYCN* amplification or del(1p); 11% were diploid with del(1p) but without *MYCN* amplification and 16% had both *MYCN* amplification and del(1p). A further 13% had other changes but without del(1p) or *MYCN* amplification.

The incidence of *MYCN* amplification in primary neuroblastoma has been quoted as around 38% in stage 3 and 4 tumours, and

© 2000 Cancer Research Campaign

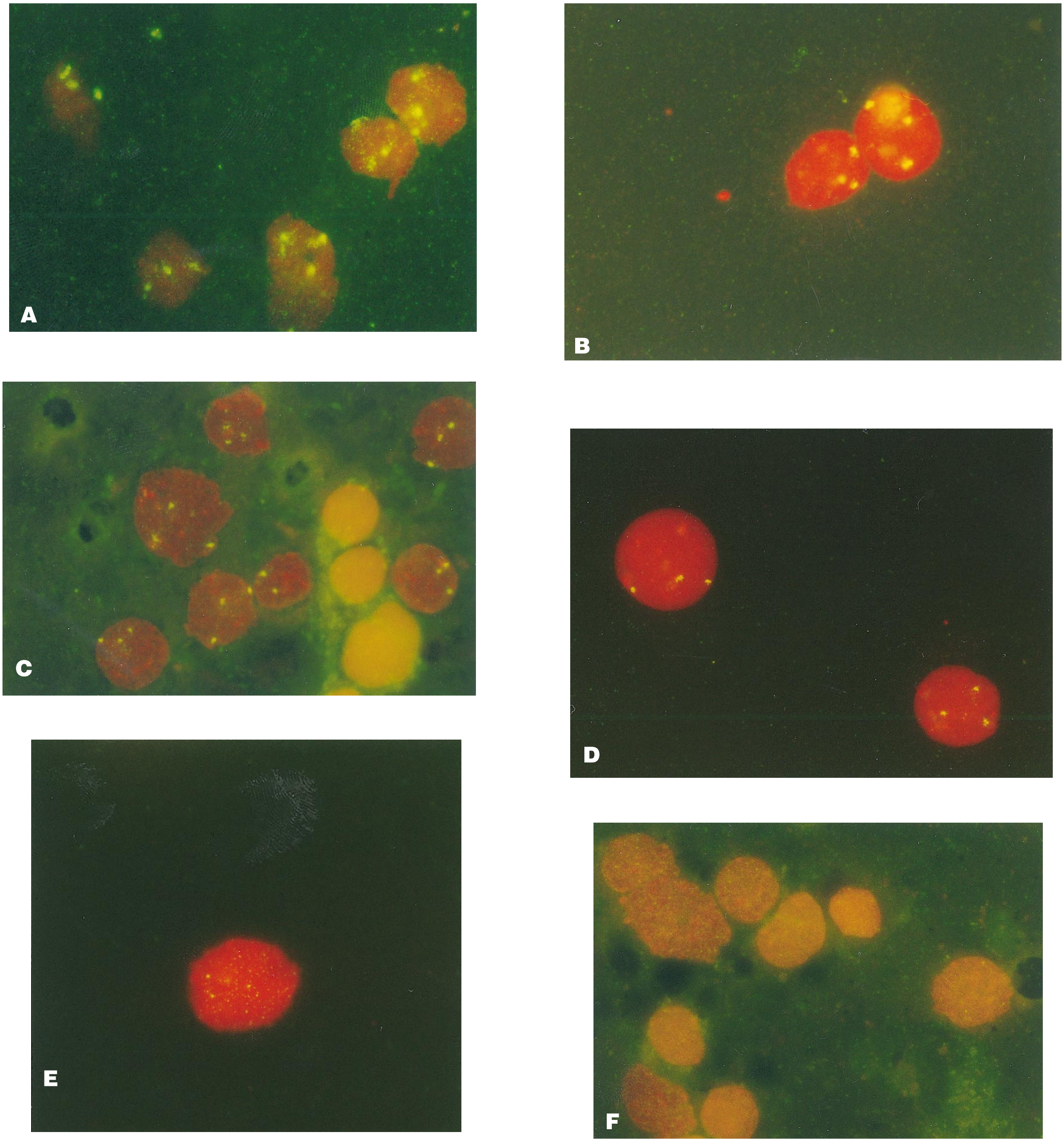

**Figure 4** (**A**) Chromosome 1 cent.-imprint; (**B**) Chromosome 1 cent.-direct preparation; (**C**) chromosome 8 cent.-imprint; (**D**) Chromosome 8 cent.-direct preparation; (**E**) CT4-1-direct preparation; (**F**) *MYCN*-imprint. Photographs showing the difference between a tumour imprint and a direct nuclear preparation (case 24). The direct preparation is much cleaner with no red cells or other debris on the slide. Both sets of results show three copies of each probe, implying triploidy, with no *MYCN* amplification

5–10% in stages 1,2 and 4S (Brodeur et al, 1984; Brodeur and Fong, 1989). Our finding of 16% is in keeping with results in other studies: 17% of 12 tumours (Shapiro et al, 1993), 15% of 59 tumours (Bourhis et al, 1991*a*) and 25% of 147 tumours (Look et al, 1991). In a series of 316 consecutive cases from the French NBL 90 study, *MYCN* amplification was detected by Southern blotting in 10% of 225 children tested (Rubie et al, 1997). Similarly our finding of 34% apparently triploid cases is in keeping with Bourhis et al (1991) (47%) and Oppedal et al (1988) (34%).

Molecular techniques detect chromosome 1p deletion in 30–40% of tumours (Fong et al, 1992; Takayama et al, 1992; Caron et al, 1993; Schleiermacher et al, 1994), although there are lower estimates, of 20% (Takeda et al, 1994) and 27% (Peter et et al, 1992). Rubie et al (1997) found an incidence of LOH1p of 10%

 © 2000 Cancer Research Campaign in 91 cases attempted. In this study the incidence of del(1p) was 26% and the incidence of *MYCN* amplification was 16%. The cases with *MYCN* amplification were a subset (60%) of those with del(1p). Comparable findings have been reported in other series, in that all cases with *MYCN* amplification also had 1p deletion, while conversely approximately 60% of cases with 1p deletion also have *MYCN* amplification (Fong et al, 1989; Caron et al, 1993).

In this study results of prognostic significance were obtained in 84% of cases, and a full set of results on all four probes in 76%. There was 100% success using direct preparations. Probes were hybridized onto separate slides with no attempt at double hybridization onto single slides, to avoid the cumulative failure rate of multiple hybridization experiments. Published data show a much lower success rate when performing FISH with more than one probe. In one study a two-probe experiment on direct preparations and cultures produced a double hybridization success rate of 38.5% (Christiansen et al, 1992).

### Controls for FISH

HFFs and neuroblastoma cell lines were used as negative and positive controls. The signals in 600 nuclei were counted for each of the probes in the study. The least efficient probe on the HFF preparations was the chromosome 1 centromere, pUC1.77. This showed two discrete signals in 78% of nuclei, no signal in 2%, one signal in 14%, three signals in 3% and four signals in 3%. A similar figure (75.8%) is given for control experiments with the same probe in another study (Christiansen et al, 1992). It was decided that concordance of signal number in at least 70% of nuclei (whether on imprints or cultured cells) was required to be able to state the number of copies present reliably. Conversely, sub-populations with different copy numbers needed to comprise at least 30% of the cell population, so a biclonal tumour needed to have two populations each over 30% of the total. This stipulation may have meant that minor sub-clones were not recorded, but this is unlikely to be a consideration of clinical importance as the genetic aberrations of prognostic significance are very consistent.

### Detection of MYCN

Results were obtained using FISH with the *MYCN* probe in 84% of cases. Samples were sent for Southern blotting in 55% of patients in the study, and results were obtained in 47% of patients. A similar proportion (49%) were sent for blotting in a much larger series of 298 patients, with results being obtained in almost all of these (48%) (Look et al, 1991). However no alternative method of *MYCN* copy number estimation was being studied. A study comparing FISH results for *MYCN* in neuroblastoma cell lines with Southern blotting results found full correlation, with 13 of the 20 cell lines showing amplification of *MYCN* with both FISH and blotting (Shapiro et al, 1993). However no blotting results were available for any of the 12 primary tumour samples evaluated, of which two (17%) were found to have *MYCN* amplification using FISH.

In our study ten neuroblastoma cell lines were evaluated with FISH of which four had known amplification of *MYCN*. There was full correlation of FISH estimates of *MYCN* copy number with available cell line data. In our study both Southern blotting and FISH results were available in 22 patients. Of these 18 were negative for *MYCN* amplification both with FISH and blotting. Four had amplification of *MYCN* using FISH and three had amplification on Southern blotting. In these three the copy number estimate from blotting was consistently lower than that from FISH, which may be because of admixture of the tumour with normal stromal tissue (Shapiro et al, 1993). In the discrepant case the appearance of the amplification was not typical, in that there was intercellular uniformity of *MYCN* copy number. This case appeared early in the series and with experience the unusually coarse, bright background fluorescence would not have been interpreted as *MYCN* amplification.

The correlation between FISH and Southern blotting in this study is therefore reduced to 95%, which still compares well with other reports. A similar study using FISH and Southern blotting to detect ERBB2 amplification in primary breast tumours detected amplification in ten of 44 tumours using interphase FISH, but in only eight using a slot blot technique (Kallioniemi et al, 1992). In a study of 23 neuroblastomas which were all carrying *MYCN* amplification by Southern blotting, a 96% correlation was found with PCR (Crabbe et al, 1992). A more recent report comparing the FISH with semi-quantitative PCR for detection of *MYCN* amplification in neuroblastoma cell lines found that FISH could detect one cell in 1000 normal cells, whilst PCR required 10% tumour cells to reliably detect the amplification (Eckschlager and McClain, 1996)

### Ploidy

The detection of ploidy in this study has been based on the assessment of two centromere probes, plus, in cases without *MYCN* amplification, a count of the number of copies of the *MYCN* probe in its normal position on chromosome 2. This method provides an adequate guide to ploidy level in the majority of cases. For absolute certainty of ploidy in any sample one would need to count centromere signals from the majority of chromosomes. This issue has not been addressed much in the literature; one paper states that ‘usually three or more chromosome specific probes have to be used to estimate the ploidy of a tissue by NISH on interphase nuclei’ (Stock et al, 1993). On this basis the three chromosomes assessed in this study would be regarded as sufficient.

### CONCLUSION

This study has shown the utility of fluorescence in situ hybridization in the detection of *MYCN* amplification, ploidy and 1p deletions in tumour imprints, bone marrow smears and direct nuclear preparations from neuroblastomas. The pilot study (Taylor et al, 1994) was among the first to describe the use of these types of preparation for FISH and was also the first publication describing visualisation of these three clinically significant genetic prognostic factors in neuroblastoma. This new study is now the largest series of patients in whom FISH has been used in this way. Other studies have evaluated only one of the clinically important genetic aberrations. One series reports on 20 patients in whom *MYCN* amplification was assessed by FISH and by Southern blotting together with karyotyping. Concordant results were obtained in the 14 fully evaluable patients (Avet-Loiseau et al, 1995). Another study successfully evaluated 1p deletion by FISH and RFLP analysis in 8/9 neuroblastoma cell lines and 23/28 patient samples (Combaret et al, 1995). Both authors felt that FISH offered many advantages over conventional molecular approaches. A series of 54 stage 1, 2 and 4S patients has been studied using FISH, cytogenetics, Southern blotting and PCR to detect del(1p), *MYCN* amplification

© 2000 Cancer Research Campaign and ploidy (Ambros et al, 1995). However, the low level of positive results in these patients and the absence of any control data makes this report difficult to interpret.

The importance of various biological factors as markers of aggressiveness of the disease has been recognized for some time, and measurement of these variables has increasingly been carried out in the clinical trial setting. The agreed goal of the International Neuroblastoma Staging System and Response Criteria Committee is to collect information on tumour histology, ploidy, *MYCN* gene copy number, chromosome 1p deletion and serum concentrations of neurone specific enolase, ferritin and lactate dehydrogenase for all neuroblastomas (Brodeur et al, 1993; Castleberry et al, 1997). Analysis of these data will indicate which biological features are sufficiently discriminatory to form the basis of therapeutic decisions. Results of the investigations into *MYCN* amplification, ploidy and 1p deletion need to be available at the time of diagnosis and commencement of treatment. The chosen methodology needs to produce reliable, rapid and reproducible results. Other methods of evaluating these biological factors require more tissue than is frequently obtained, plus expensive or specialized techniques that are not readily available in all centres. Our fast FISH method has now been thoroughly evaluated. It is able to produce reliable results in a much higher proportion of patients (84%) than conventional methods. It is therefore appropriate that this application of FISH should become accessible to all clinicians practising in this field. The technique is simple, and, unlike complex cytogenetic and molecular biological studies, could be performed rapidly either in a national reference centre or in routine service laboratories without the need for complex and expensive computerised imaging techniques.

### ACKNOWLEDGEMENTS

The authors thank the oncologists, surgeons, pathologists and histopathology staff at The Royal Victoria Infirmary, Newcastle, and The Hospital for Sick Children, Great Ormond Street, London, in particular Dr Jon Pritchard, Consultant Oncologist, GOS. We are also indebted to Dr Manfred Schwab, Heidelberg, Germany, for the pNb-9 and p1-24 plasmids; Dr M Rocchi and Dr A Baldini for permission to use the chromosome 8 centromere probe, and Dr J Kemshead for the neuroblastoma cell lines. We are very grateful to the Emma Killingback Memorial Fund and the NE England Children's Cancer Fund for crucial financial support.

### REFERENCES

Ambros PF, Ambros IM, Strehl S, Bauer S, Luegmayr A, Kovar H, Ladenstein R, Fink FM, Horcher E, Printz G, Mutz I, Schilling F, Urban C and Gadner (1995) Regression and progression in neuroblastoma. Does genetics predict tumour behaviour? *Eur J Cancer* **31A**: 410–515

Avet-Loiseau H, Venaut AM, Benard J, Leibovitch MP, Hartmann O and Bernheim A (1995) Morphologic and molecular cytogenetics in neuroblastoma. *Cancer* **75**: 1694–1699

Bourhis J, DeVathaire F, Wilson GD, Hartmann O, Terrier-Lacombe MJ, Boccon-Gibod L, McNally NJ, Lemerle J, Riou G and Benard J (1991*a*) Combined analysis of DNA ploidy index and N-myc genomic content in neuroblastoma. *Cancer Res* **52**: 33–36

Bourhis J, Dominici C, McDowell H, Raschella G, Wilson G, Castello MA, Plouvier E, Lemerle J, Riou G, Benard J and Hartmann O (1991*b*) N-myc genomic content and DNA ploidy in stage IVs neuroblastoma. *J Clin Oncol* **9**: 1371–1375

Bown N, Cotterill S, Lastowska M, Brinkschmidt C, Meddeb M, Plantaz D and Speleman F (1998) European multicentre study of 274 neuroblastoma tumours demonstrates that 17q gain is a powerful prognostic indicator. *Med Ped Oncol* **31**: 190

Bown N, Cotterill S, Lastowska M, O'Neill S, Pearson AD, Plantaz D, Meddeb M, Danglot G, Brinkschmidt C, Christiansen H, Laureys G and Speleman F (1999) Gain of chromosome arm 17q and adverse outcome in patients with neuroblastoma. *N Engl J Med* **340**: 1954–1961

Brodeur GM and Fong C-T (1989) Molecular biology and genetics of human neuroblastoma. *Cancer Genet Cytogenet* **41**: 153–174

Brodeur GM, Seeger RC, Schwab M, Varmus HE and Bishop JM (1984) Amplification of N-myc in untreated human neuroblastomas correlates with advanced disease stage. *Science* **224**: 1121–1124

Brodeur GM, Pritchard J, Berthold F, Carlsen NLT, Castel V, Castleberry, RP, De Bernardi B, Evans AE, Favrot M, Hedborg F, Kaneko M, Kemshead J, Lampert F, Lee REJ, Look AT, Pearson ADJ, Philip T, Roald B, Sawada T, Seeger RC, Tsuchida Y and Voute PA (1993) Revisions of the international criteria for neuroblastoma diagnosis, staging and response to treatment. *J Clin Oncol* **11**: 1466–1477

Caron H, van Sluis P, van Hoeve M, de Kraker J, Bras J, Slater R, Mannens M, Voute PA, Westerveld A and Versteeg R (1993) Allelic loss of chromosome 1p36 in neuroblastoma is of preferential maternal origin with N-myc amplification. *Nature genetics* **4**: 187–190

Caron H, van Sluis P, de Kraker J, Bokkerink J, Egeler M, Laureys G, Slater R, Westerveld A, Voute PA and Versteeg R (1996) Allelic loss of chromosome 1p as a predictor of unfavourable outcome in petients with neuroblastoma. *N Engl J Med* **334**: 225–230

Castleberry RP, Pritchard J, Ambros P, Berthold F, Brodeur GM, Castel V, Cohn SL, De Bernardi B, Dicks-Mireaux C, Frappaz D, Haase GM, Haber M, Jones DR, Joshi VV, Kaneko M, Kemshead JT, Kogner P, Lee REJ, Matthay KK, Michon JM, Monclair R, Roald BR, Seeger RC, Shaw PJ, Shimada H and Shuster JJ (1997) The international neuroblastoma report. *Eur J Cancer* **33**: 2116–2116

Christiansen H and Lampert F (1988) Tumour karyotype discriminates between good and bad prognostic outcome in neuroblastoma. *Br J Cancer* **57**: 121–126

Christiansen H, Schestag J, Christiansen NM, Grzeschik K-H and Lampert F (1992) Clinical impact of chromosome 1 aberrations in neuroblastoma: a metaphase and interphase cytogenetic study. *Genes, Chromosomes Cancer* **5**: 141–149

Combaret V, Turc-Carel C, Thiesse P, Rebillard AC, Frappaz D, Haus O, Philip T and Favrot MC (1995) Sensitive detection of numerical and structural aberrations of chromosome 1 in neuroblastoma by interphase fluorescence in situ hybridization. Comparison with restriction fragment length polymorphism and conventional cytogenetic analysis. *Int J Cancer* **61**: 185–191

Cooke HJ and Hindley J (1979) Cloning of human satellite III DNA: Different components are on different chromosomes. *Nucleic Acids Res* **6**: 3177–3197

Crabbe DCG, Peters J and Seeger RC (1992) Rapid detection of MYCN gene amplification in neuroblastoma using the polymerase chain reaction. *Diagn Mol Pathol* **1**: 229–234

Eckschlager T and McClain K (1996) Comparison of fluorescent in situ hybridization (FISH) and the polymerase chain reaction (PCR) for the detection of residual neuroblastoma cells. *Neoplasma* **43**: 301–303

Evans AE, D'angio GJ, Propert K, Anderson J and Hann H-WL (1987) Prognostic factors in neuroblastoma. *Cancer* **59**: 1853–1859

Fong C, Dracopoli NC, White PS, Merrill PT, Griffith RC, Housman DE and Brodeur GM (1989) Loss of heterozygosity for the short arm of chromosome 1 in human neuroblastomas: correlation with N-myc ampification. *Proc Natl Acad Sci USA* **86**: 3753–3757

Fong CT, White PS, Peterson K, Sapienza C, Cavenee WK, Kern S, Vogelstein B, Cantor AB, Look AT and Brodeur GM (1992) Loss of heterozygosity for chromosome 1 or 14 defines subsets of advanced neuroblastomas. *Cancer Res* **52**: 1780–1785

Gansler T, Chatten J, Varello M, Bunin GR and Atkinson B (1986) Flow cytometric analysis of neuroblastoma: correlation with histology and clinical outcome. *Cancer* **58**: 2453–2458

Hayashi Y, Kanda N, Inaba T, Hanada R, Nagahara N, Muchi H and Yamamoto K (1989) Cytogenetic findings and prognosis in neuroblastoma with emphasis on marker chromosome 1. *Cancer* **63**: 126–132

Kallioniemi O-P, Kallioniemi A, Kurisu W, Thor A, Chen L-C, Smith HS, Waldman FM, Pinkel D and Gray JW (1992) ERBB2 amplification in breast cancer analysed by fluorescence in situ hybridization. *Proc Natl Acad Sci USA* **89**: 5321–5325

Lastowska M, Nacheva E, McGuckin A, Curtis A, Grace C, Pearson A and Bown N (1997) Comparative genomic hybridization study of primary neuroblastoma tumours. United Kingdom Children's Cancer Study Group. *Genes Chromosomes Cancer* **18**: 162–169

 *© 2000 Cancer Research Campaign*

Look AT, Hayes FA, Nitschke R, McWilliam N and Green A (1984) Cellular DNA content as a predictor of response to chemotherapy in infants with unresectable neuroblastoma. *N Engl J Med* **311**: 231–235

Look AT, Hayes FA, Shuster JJ, Douglass EC, Castleberry RP, Bowman LC, Smith EI and Brodeur GM (1991) Clinical relevance of tumor cell ploidy and N-myc gene amplification in childhood neuroblastoma: a pediatric oncology group study. *J Clin Oncol* **9**: 581–591

Meddeb M, Danglot G, Chudoba I, Venuat A-M, Benard J, Avet-Loiseau H, Vassear B, Le Paslier D, Terrier-Lacombe M-J, Hartmann O and Bernheim A (1996) Additional copies of a 25Mb chromosomal region originating from 17q23.1–17qter are present in 90% of high grade neuroblastomas. *Genes Chromosomes Cancer* **17**: 156–165

Oppedal BR, Storm-Mathisen I, Lie SO and Brandtzaeg P (1988) Prognostic factors in neuroblastoma. Clinical, histopathologic, and immunohistochemical features and DNA ploidy in relation to prognosis. *Cancer* **62**: 772–780

Peter M, Michon J, Vielh P, Neuenschwander S, Nakamura Y, Sonsino E, Zucker J-M, Vergnaud G, Thomas G and Delattre O (1992) PCR assay for chromosome 1p deletion in small neuroblastoma samples. *Int J Cancer* **52**: 544–548

Pinkel D, Shaume T and Grey JW (1986) Cytogenetic analysis using quantitative, high sensitivity, fluorescence hybridization. *Proc Natl Acad Sci USA* **83**: 2934

Rubie H, Delattre O, Hartmann O, Combaret V, Michon J, Benard J, Peyroulet MC, Plantaz D, Coze C, Chastagner P, Baranzelli MC, Frappaz D, Lemerle J and Sommelet D (1997) Loss of chromosome 1p may have a prognostic value in localised neuroblastoma: results of the French NBL 90 study. *Eur J Cancer* **33**: 1917–1922

Schleiermacher G, Peter M, Michon J, Hugot J-P, Vielh P, Zucker J-M, Magdelenat H, Thomas G and Delattre O (1994) Two distinct deleted regions on the short arm of chromosome 1 in neuroblastoma. *Genes Chromosomes Cancer* **10**: 275–281

Schwab M, Alitalo K, Klempnauer K-H, Varmus HE, Bishop JM, Gilbert F, Brodeur GM, Goldstein M and Trent J (1983) Amplified DNA with limited homology to myc cellular oncogene is shared by human neuroblastoma cell lines and a neuroblastoma tumour. *Nature* **305**: 245–248

Schwab M, Varmus HE, Bishop JM, Grzeschik K-H, Naylor SL, Sakaguchi AY, Brodeur GM and Trent J (1984) Chromosome localization in normal human cells and neuroblastomas of a gene related to c-myc. *Nature* **308**: 288–291.

Seeger RC, Brodeur GM, Sather H, Dalton A, Siegel SE, Wong KY and Hammond D (1985) Association of multiple copies of the N-myc oncogene with rapid progression of neuroblastomas. *N Engl J Med* **313**: 1111–1116

Shapiro DN, Valentine MB, Rowe ST, Sinclair AE, Sublett JE, Roberts WM and Look AT (1993) Detection of N-myc gene amplification by fluorescence in situ hybridization: diagnostic utility for neuroblastoma. *Am J Pathol* **142**: 1339–1346

Shimada H, Chatten J, Newton WA, Sachs N, Hamoudi AB, Chiba T, Marsden HB and Misuki K (1984) Histopathologic prognostic factors in neuroblastic tumors: definition of subtypes of ganglio-neuroblastomas and age linked classification of neuroblastomas. *J Natl Cancer Inst* **73**: 405–416

Stock C, Ambros AM, Mann G, Gadner H, Amann G and Ambros P (1993) Detection of 1p36 deletions in paraffin sections of neuroblastoma tissues. *Genes Chromsomes Cancer* **6**: 1–9

Takayama H, Suzuki T, Mugishima H, Fujisawa T, Ookuni M, Schwab M, Gehring M, Nakamura Y, Sugimura T, Terada M and Yokota J (1992) Deletion mapping of chromosomes 14q and 1p in human neuroblastoma. *Oncogene* **7**: 1185–1189

Takeda O, Homma C, Maseki N, Sakurai M, Kanda N, Schwab M, Nakamura Y and Kaneko Y (1994) There may be two tumor suppressor genes in chromosome arm 1p closely associated with biologically distinct subtypes of neuroblastoma. *Genes*, *Chromosomes and Cancer* **10**: 30–39

Taylor CPF, McGuckin AG, Bown NP, Reid MM, Malcolm AJ, Pearson ADJ and Sheer D (1994) Rapid detection of prognostic genetic factors in neuroblastoma using fluorescence in situ hybridisation on tumour imprints and bone marrow smears. *Br J Cancer* **69**: 445–451

Taylor SR, Blatt J, Constantino JP, Roederer M and Murphy RF (1988) Flow cytometric analysis of neuroblastoma and ganglioneuroma: a 10 year retrospective study. *Cancer* **62**: 749–754

Taylor SR and Locker J (1990) A comparative analysis of nuclear DNA content and N-myc gene amplification in neuroblastoma. *Cancer* **65**: 1360–1366

Tsuda T, Obara M, Hirano H, Gotoh S, Kubomura S, Higashi K, Kuroiwa A, Nakagawara A, Nagahara N and Shimizu K (1987) Analysis of N-myc amplification in relation to disease stage and histologic types in human neuroblastomas. *Cancer* **60**: 820–826

Weith A, Martinsson T, Cziepluch C, Bruderlein S, Amler LC, Berthold F and Schwab M (1989) Neuroblastoma consensus deletion maps to 1p36.1-2. *Genes Chromosomes Cancer* **1**: 159–166

White PS, Kaufman BA, Marshall HN and Brodeur GM (1993) Use of the single-strand conformation polymorphism technique to detect loss of heterozygosity in neuroblastoma. *Genes Chromosomes Cancer* **7**: 102–108

*© 2000 Cancer Research Campaign* 
